# From neurotransmitters to networks: Transcending organisational hierarchies with molecular-informed functional imaging

**DOI:** 10.1016/j.neubiorev.2023.105193

**Published:** 2023-07

**Authors:** Timothy Lawn, Matthew A. Howard, Federico Turkheimer, Bratislav Misic, Gustavo Deco, Daniel Martins, Ottavia Dipasquale

**Affiliations:** a Department of Neuroimaging, Institute of Psychiatry, Psychology and Neuroscience, King's College London, London, UK; b Montreal Neurological Institute and Hospital, McGill University, Montreal, Québec, Canada; c Center for Brain and Cognition, Computational Neuroscience Group, Department of Information and Communication Technologies, Universitat Pompeu Fabra, Ramon Trias Fargas 25-27, Barcelona 08005, Spain; d Department of Neuropsychology, Max Planck Institute for Human Cognitive and Brain Sciences, Leipzig, Germany; e Institució Catalana de Recerca i Estudis Avançats (ICREA), Barcelona, Spain; f Turner Institute for Brain and Mental Health, Monash University, Melbourne, VIC, Australia

**Keywords:** FMRI, Multimodal, PET, Imaging, Transcriptomic, AHBA, Expression, Molecular, Receptor, REACT, Network, Functional connectivity

## Abstract

The human brain exhibits complex interactions across micro, meso-, and macro-scale organisational principles. Recent synergistic multi-modal approaches have begun to link micro-scale information to systems level dynamics, transcending organisational hierarchies and offering novel perspectives into the brain’s function and dysfunction. Specifically, the distribution of micro-scale properties (such as receptor density or gene expression) can be mapped onto macro-scale measures from functional MRI to provide novel neurobiological insights. Methodological approaches to enrich functional imaging analyses with molecular information are rapidly evolving, with several streams of research having developed relatively independently, each offering unique potential to explore the trans-hierarchical functioning of the brain. Here, we address the three principal streams of research – spatial correlation, molecular-enriched network, and *in-silico* whole brain modelling analyses – to provide a critical overview of the different sources of molecular information, how this information can be utilised within analyses of fMRI data, the merits and pitfalls of each methodology, and, through the use of key examples, highlight their promise to shed new light on key domains of neuroscientific inquiry.

## Highlights

• The brain exhibits micro-, meso-, and macro-scale organisational principles.
• The BOLD signal is blind to the molecular mechanisms that give rise to it.
• fMRI data can be mapped onto molecular systems derived in normative cohorts.
• Approaches to link fMRI and molecular data each offer distinct pros and cons.
• Molecular-functional relationships provide biological interpretability to fMRI analyses.

## 1 Introduction

Neuropsychiatric disorders present a formidable healthcare challenge for which we remain largely bereft of targeted and mechanistically informed treatments. Despite substantial progress in recent decades, this largely reflects our current inability to delineate the elusive fundamental principles governing brain function. This is due in part to the challenges presented by the hierarchical organisation of the brain, which exhibits complex non-linear interactions across micro-, meso- and macro-scale systems (Box 1). Herein, we use the term hierarchical to refer to this multi-scale organisation (Hilgetag and Goulas, 2020) integrating nested and increasingly polyfunctional elements, which ultimately subserve behaviour and cognition (Suárez et al., 2020). To date, efforts to unravel this complexity have mostly followed a reductionist approach within which each organisational scale is explored independently. For example, cognitive neuroscience has benefited enormously from the advent and expansion of non-invasive imaging techniques exploring neural mechanisms at the macro-scale systems level (Bassett et al., 2020). In this context, functional magnetic resonance imaging (fMRI) has become the principal tool in human research to map mental processes to their neurobiological substrates, characterize dysfunction of the brain in a variety of clinical conditions, and study the brain's response to pharmacological challenges. However, the utility of the blood oxygen level dependent (BOLD) signal in addressing these core neuroscientific questions is constrained by its indirect nature and inherent inability to provide information as to the cellular and molecular processes that give rise to it. Together, these limitations leave BOLD fMRI practically and conceptually detached from domains of neuroscientific inquiry that explore the fine-grain biochemical basis of brain function and dysfunction.

Box 1 **The hierarchical micro-, macro-, and meso-scale organisation of the brain.**

The brain is a complex system whose constitutive parts span vastly different spatial resolutions. Here, we describe these as a hierarchy with interactions across micro-, meso-, and macro-scale levels. The definitions of these levels are somewhat arbitrary, but this loose demarcation has proven conceptually useful (Fornito et al., 2019a, Fornito et al., 2019b, Suárez et al., 2020, Swanson and Lichtman, 2016, van den Heuvel et al., 2019, Wong-Lin et al., 2021). Firstly, at the micro-scale, molecular processes including − but far from limited to − genetics, transcriptomics, and proteomics delineate the morphology and dynamics of neurones. The cytoarchitectonic arrangement of these neurones into circuits constitutes the meso-scale, a somewhat underappreciated link between micro-scale processes and macro-scale dynamics. Finally, the macro-scale captures how the brain’s network architecture emerges from the activity across regions, collectively constituting larger systems. Crucially, there are interactions across the different levels of this hierarchical organisation of the brain, which need not be limited to the relatively straightforward example of lower-level features building up to constitute higher-level features. Minute changes in the regional concentrations of neuromodulatory transmitter systems can enact complex downstream effects through metabotropic receptors at the micro-scale, which can result in drastic non-linear effects on emerging network dynamics at the macro-scale (Shine, 2019). Additionally, one scale can constrain another, with regions showing similar cytoarchitecture or gene expression potentially showing greater propensity to interact and connect, resulting in a constraint on the patterns of emerging macro-scale connectivity between regions (van den Heuvel and Yeo, 2017). Conversely, the pattern of macro-scale connectivity may constrain the spread of micro-scale neuropathology, such as misfolded proteins, throughout the brain (van den Heuvel and Yeo, 2017, Zhou et al., 2012). The methods outlined herein offer novel tools to explore such relationships and build unified theories of brain function that span the different levels of this organisation hierarchy.

.

Developing comprehensive accounts of the brain and its disorders must therefore bridge the theoretical void between the micro- and macro-scale organisation of the nervous system. The human brain shows rich variations in myelo-, cyto-, and, crucially, chemo-architecture (van den Heuvel and Yeo, 2017). Indeed, recent evidence has shown that neuroreceptor densities demonstrate a natural axis of spatial organization through the cortex, with links to the laminar as well as the functional hierarchical organisation of the cortical mantle (Goulas et al., 2021). This information can be leveraged through mapping particular micro- and meso-scale properties of the human brain onto its macro-scale haemodynamics, providing neurobiological specificity to the BOLD signal and generating novel hypotheses which transcend organisational hierarchies. Specifically, these approaches utilise molecular information, such as neurotransmitter systems or expression of genes, to examine spatial covariation with measures of brain activation or connectivity. This has primarily been made possible by the expansion and public availability of receptor density atlases from Positron Emission Tomography (PET) and Single-Photon Emission Computed Tomography (SPECT), as well as transcriptomic data from sources such as the Allen Human Brain Atlas (AHBA)(Hawrylycz et al., 2015), which have provided an impetus for novel multimodal analyses that exploit this molecular information to enrich conventional fMRI methods. At its simplest, this involves considering the spatial concordance between patterns of resting state functional connectivity (FC) or task-based activation and the distribution of different receptor densities or expressed genes (Arnatkevic̆iūtė et al., 2019, Fornito et al., 2019a, Fornito et al., 2019b, Richiardi et al., 2015, Selvaggi et al., 2021). Other approaches have delineated more complex spatiotemporal relationships. For example, Receptor-Enriched Analysis of functional Connectivity by Targets (REACT) was recently developed to map the spatiotemporal dynamics of the BOLD signal onto the distribution of different receptor systems and derive receptor-enriched networks that link biology and FC (Dipasquale et al., 2019). Additionally, computational approaches such as *in silico* modelling of whole brain dynamics have been used to explore the putative contribution of receptor subsystems to cortical dynamics (Jancke et al., 2021). These novel integrative methods, that bring together micro-scale information at the molecular level with macro-scale neuroimaging features, have shown initial promise in helping characterise the brain’s i) functional network architecture and its relationship to cognition, ii) perturbation within disease, and iii) response to drugs. Crucially, pharmacotherapy is the mainstay of neuropsychiatric treatment, and network-level dysfunction characterised using fMRI remains abstracted from the molecular-level mechanisms through which these interventions impart their benefit. Through understanding the disorder- and subject-specific dysfunction of the molecular systems underlying disease, we may be able to target treatment through links to the known pharmacology and pharmacodynamic effects of drugs, bringing theoretical knowledge gained from functional neuroimaging closer to the needs of patients.

This field is now gathering significant momentum, with several recent papers showcasing advances in our understanding of the brain by linking molecular information to its inherent functional organisation (Hansen et al., 2022a), cognition (Hansen et al., 2022a, Hansen et al., 2021b), disease states (Hansen et al., 2022b), and psychopharmacology (Luppi et al., 2022a). In this narrative review, we will explore the applications and promises of synergistic multimodal analysis of fMRI data. First, we provide an overview of the sources of molecular information and their respective use cases. Next, we critically outline each of the three main methods employed within synergistic multimodal research, including their respective strengths and weaknesses (overviewed in Table 1). Alongside these methods, we highlight examples of their applications to various domains of neuroscientific inquiry, here used to discuss how multimodal neuroimaging analyses have advanced our understanding of the brain and its perturbation in disease.

**Table 1:** Methodologies incorporating molecular information into the analysis of fMRI data. PET: Positron Emission Tomography; AHBA: Allen Human Brain Atlas; PLS: Partial Least Squares regression.

**Method**	**Summary**	**Types of molecular information**	**Advantages**	**Disadvantages**	**Key applications**
Spatial correlation (Section 3)	Conventional analysis of fMRI data is undertaken and then resulting un-thresholded maps are correlated with the spatial distribution of molecular information	• Receptor/transporter density (PET) • Transcriptomics (AHBA) • Cytoarchitectonics	• Simple • Flexible • Easily interpretable • Opportunity to scale up (PLS)	• Less insight into spatiotemporal dynamics • Less amenable to providing subject-specific information • Less clear spatial localisation	• Adding as secondary outcome to provide biological specificity • Large scale mapping of molecular-functional relationships (PLS)
Molecular-enriched networks (Section 4)	A multiple regression framework creates functional networks capturing relationships between BOLD signal and spatial distribution of molecular information	• Receptor/transporter density (PET) • Transcriptomics (AHBA)	• Spatiotemporal insight • Can apply conventional higher level analyses	• Interpretation of negative functional connectivity can be challenging • Collinearity across PET templates requires careful consideration	• Disentangling pharmacodynamics • Novel biomarkers
Computational modelling (Section 5)	Creation of whole brain models that attempt to recapitulate experimental data from functional imaging, within which molecular information can be used to regionally modulate different aspects of neuronal micro-circuitry and examine the counterfactual consequences	• Receptor/transporter density (PET) • Transcriptomics (AHBA)	• Requires careful hypotheses • Allows for manipulation and mechanistic insight not possible experimentally	• Technically challenging • Resource intensive • Modelling multiple receptor systems and their interactions is currently difficult	• Testing hypotheses regarding the role molecular mechanisms play in shaping network dynamics • Characterising contribution of different receptor mechanisms to drug pharmacodynamics

## 2 Sources of molecular information

A key commonality of studies enriching fMRI with molecular information is the requirement for high-quality data delineating the distribution of receptors and expression of genes. To date, these have come primarily from two sources. Firstly, gradual progress in PET and SPECT tracer development, as well as data-sharing practices, have resulted in a broad range of openly available and shared atlases providing voxel-wise estimates of receptor density averaged across healthy participants (Dukart et al., 2021, Hansen et al., 2022a, Knudsen et al., 2020, Nørgaard et al., 2021, Tuominen et al., 2014). These include retrospectively shared data from ongoing applied PET/SPECT research, but also a high-resolution multi-receptor serotonergic atlas derived in a very large (N = 210) normative sample (Beliveau et al., 2017), which marks an important step towards mapping the brain’s ‘receptome’ (Jancke et al., 2021). Secondly, large-scale projects such as the AHBA provide a rich characterisation of expression patterns with > 20,000 genes across 3702 tissue samples measured within 6 adult neurotypical human brains (Hawrylycz et al., 2015)(for detailed review, see (Arnatkevic̆iūtė et al., 2019)). EBRAINS (https://ebrains.eu/) also offers a Multilevel Human Brain Atlas which provides various micro- and meso-scale facets of brain organisation integrated into a high-resolution atlas. However, other sources exist. Autoradiography mapping has largely been limited by poor whole brain anatomical coverage (Zilles and Palomero-Gallagher, 2017). Similarly, the Genotype-Tissue Expression (GTEx) project offers an additional source of transcriptional information in a larger cohort than the AHBA, but only from a select few regions (Aguet et al., 1979). Accordingly, the primary focus is on PET estimates of receptor density and AHBA measures of gene expression, both of which can be mapped onto the same three-dimensional anatomical space as fMRI data, allowing for straightforward applications within novel multimodal analyses.

### 2.1 Selecting a source: binding potential or gene expression?

The choice of whether to use PET or transcriptomic molecular information is largely contingent on the research question. For instance, the AHBA offers a vast array of expression data covering the full breadth of the transcriptome, whilst PET data remains constrained to the receptor systems and sub-systems for which ligands have been developed. As such, approaches exploring a wide range of molecular systems, or analyses of specific systems for which no PET tracer is available, favour the breadth of the AHBA. PET data generally provides superior spatial resolution, allowing for voxel-wise rather than parcellated region of interest (ROI)-based analyses, although recent attempts have been made to create vertex/voxel-wise maps of gene expression using machine learning (Gryglewski et al., 2018; Markello et al., 2021; Wagstyl et al., 2022)). Furthermore, the limited sample of six individuals, of which only two have both hemispheres sampled, calls into question the generalisability of the AHBA data. Moreover, transcriptomic data has the potential limitations that mRNA only approximates actual protein levels (due to post-transcriptional regulation, transcript isoforms, splice variant expression, and protein buffering) as well as the mismatch between where the mRNA is produced (the soma) and where many proteins are expressed (for example, most receptors are located at the synapse). The extent of these limitations varies between receptor systems – e.g., while the 5-HT_1a_ binding and expression are closely coupled, opioidergic receptors, which are under tighter post-translational regulation, are weakly correlated (Rizzo et al., 2014). Similarly, comparison of AHBA gene expression to protein levels measured with autoradiography demonstrated substantial variations across different receptors, with most correlations being weak (Murgaš et al., 2022). The high-resolution multi-receptor atlas of the serotonin system derived from 210 individuals has also been compared to AHBA gene expression data (Beliveau et al., 2017). Whilst the 5-HT_1a_ receptor showed excellent correspondence, it was only moderate for 5-HT_4_ and 5-HT_1b_. Moreover, the 5-HT_2a_ receptor showed only weak correlation cortically, and no correlation subcortically. The serotonin transporter showed no correlation, although both expression and binding were high in the dorsal raphe, aligning with its presynaptic localisation resulting in terminal projections transporter levels being spatially mismatched with gene expression (Beliveau et al., 2017, Hoffman et al., 1998, Zhou et al., 1998), further emphasising the nuanced relationship between these measures. More recently, a systematic comparison of 27 neurotransmitter receptors found overall poor spatial concordance between PET and AHBA data, with the exception of four metabotropic receptors (5-HT_1a_, D2, CB1, and MOR)(Hansen et al., 2021a). As such, studies enriching fMRI data with these different sources will likely produce divergent findings. Indeed, in a study trying to explain relationships between changes in cerebral blood flow using D2 receptor density and DRD2 gene expression, the former explained more variance than the latter (Selvaggi et al., 2019). However, the [18 F]-Fallypride ligand used in that study binds to D2 and D3 receptors (Hsieh et al., 2021, Slifstein et al., 2004, Vandehey et al., 2010), and thus additional explained variance could come from D3 receptor density not captured by the more specific DRD2 gene expression. Thus, extreme care must be taken regarding the specificity of findings given the large number of contributing and interacting factors underlying the distribution of receptors and their transcripts. For a more detailed discussion of theoretical and methodological challenges associated with AHBA-based analyses, see (Selvaggi et al., 2021). Altogether, from the available evidence discussed above and especially for pharmacoimaging, PET receptor density seems to offer a better estimate of receptor distribution than gene expression data (Liu et al., 2016, Selvaggi et al., 2021). In the longer term, transcriptomics may overcome some of these crucial limitations with higher resolution datasets derived from a greater number of individuals. Indeed, the extent to which genes and receptors correlate can itself be predicted from the differential stability of a gene, suggesting that improved consistency in measurement may result in better estimates of receptor abundance (Hansen et al., 2021a). In the meantime, future studies employing both sources of molecular information may allow for side-by-side comparison to help mitigate the generation and propagation of erroneous molecular-functional relationships.

### 2.2 Construct validity

A general assumption made when utilising molecular information from PET and SPECT imaging and the AHBA is that the spatial distribution of receptors derived in normative, independent cohorts is applicable to fMRI data from new samples (Dipasquale et al., 2019, Selvaggi et al., 2021). In general, the average distribution of receptor density and gene expression seems to offer useful approximations for the influence of a given receptor or gene over the BOLD signal in a given region (although see Section 2.1 for discussion of gene expression versus receptor density). This is primarily evidenced by the fact that molecular-enriched analyses to date have found hypothesis-driven pharmacodynamic effects in line with the known receptor affinity and (ant)agonist activity of different drugs (Craig et al., 2021, Dipasquale et al., 2020, Dukart et al., 2018, Lawn et al., 2023, Lawn et al., 2022, Luppi et al., 2022b, Luppi et al., 2022a; Daniel Martins et al., 2022a, Martins et al., 2022b; Preller et al., 2018; Selvaggi et al., 2019; Wong et al., 2022). However, confirmatory studies clarifying the extent to which this assumption holds will be important in the longer term. In particular, comparisons of molecular-enriched analysis conducted using PET and fMRI data from the same subjects to fMRI data collected in separate subjects may provide further insight. Moreover, the development and open sharing of PET atlases derived within large normative samples such as from Beliveau and colleagues may prove less susceptible to outliers than those from conventional PET studies, which tend to involve drastically fewer participants (Beliveau et al., 2017). Despite these potential caveats, it must also be emphasised that this molecular information provides a relatively straightforward ability to probe systems that would require prohibitively costly and invasive multi-tracer studies. Indeed, PET studies are typically conducted in under 20 participants, whilst some collaborative fMRI datasets now include over a thousand (Smith et al., 2013). Thus, whilst these techniques must be utilised with appropriate consideration of these limitations, they also provide opportunity for novel large-scale analysis of brain function.

## 3 Spatial correlation between fMRI results and molecular information

**Fig. 1:**
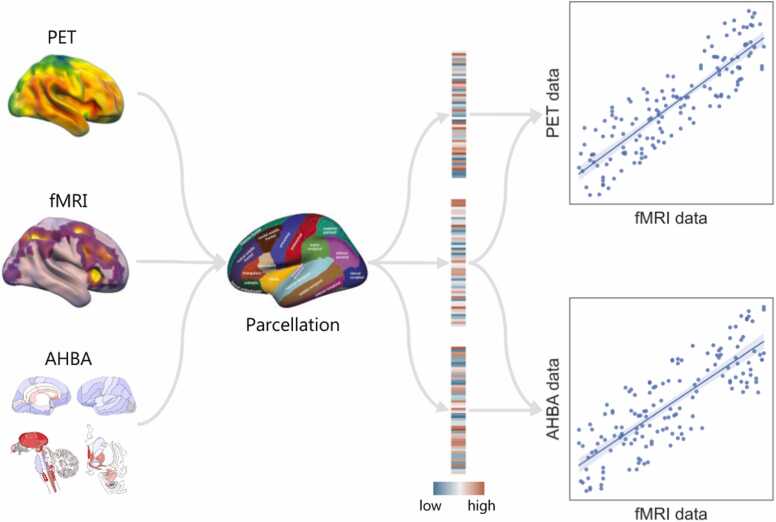
An overview of simple spatial correlation analyses. First, conventional fMRI results as well as molecular information relating to receptor systems or genes of interest from PET and/or the AHBA are parcellated using an anatomical or functional atlas, producing summary statistics of receptor binding, fMRI connectivity/activation, and AHBA gene expression (shown from top-to-bottom) within each region of the chosen parcellation. Relationships between these vectors can then be explored, typically with Pearson’s correlation coefficient, to quantify how related the patterns of fMRI connectivity/activation are to the receptor density estimates from PET (top) and/or gene expression from the AHBA (bottom).

If one assumes that a particular pattern of changes in fMRI data is driven by a certain biological process, then it is reasonable that the extent to which each region of the brain exhibits a change might be at least partially explicable by the relative abundance of the molecular machinery underlying this process across regions. The most common approach to derive molecular insights from fMRI data is to undertake conventional fMRI analyses and then examine how the results do or do not overlap with the spatial distribution of different receptors or genes of interest (Fig. 1). This approach moves away from trying to identify “where” in the brain different cognitive of pathological processes take place, towards understanding “how” these processes emerge from broader patterns of network activity. The simplicity of this method in inferring the relationships between micro-scale information at the molecular level and macro-scale neuroimaging features has led to its diverse application across many domains of neuroscience.

### 3.1 Methodology

After parcellating whole brain un-thresholded statistical maps for a contrast of interest (e.g., drug vs placebo) as well as molecular information from potentially explanatory systems (e.g., molecular density or gene expression for the receptor(s) upon which the drug acts) using an anatomical or functional atlas (e.g., the Desikan–Killiany atlas (French and Paus, 2015)), these values can be used to compute measures of correlation (typically Pearson’s correlation coefficient) between fMRI and molecular data across these brain regions. The correlation, or lack thereof, between fMRI results and a given receptor density or gene expression profile accordingly provides indirect support for or against the relevance of that molecular system for the network-level findings from the conventional fMRI analysis. While conceptually simple, correlating brain maps that inherently possess a spatially autocorrelated structure requires some additional consideration of methods which can help avoid inflated results. Since this is a longstanding issue in multimodal imaging, we cover this topic in further detail below (see Section 3.6).

The field has also moved beyond simple spatial correlation analyses and started to more broadly link multiple molecular systems to aspects of functional imaging and behaviour in a data-driven manner. When utilising multiple receptors or genes as explanatory variables to test their link with the functional measure of interest within a multiple linear regression framework to estimate the relative contribution of each variable to the total variance, one must consider that these systems frequently demonstrate strong collinearity. A commonly used technique to overcome this challenge is partial least squares regression (PLS), which reduces the number of explanatory variables to a smaller set of uncorrelated components (Krishnan et al., 2011). In this context, PLS aims to generate molecular-based components that have maximum covariance with fMRI measures. These are then ranked by their covariance such that the first few components generally provide an optimal low-dimensional representation of the covariance across the high-dimensional data. However, it must be noted that models into which multiple receptor systems are included are not a panacea, and attempts to account for collinearities generate results that are challenging to interpret (further discussed in section 4.5). Furthermore, PLS regression can also be inaccurate when the number of samples per feature is low, resulting in an inability to adequately constrain the model (Helmer et al., 2021). In particular, feature weights can be heavily biased towards the leading principal component axes, which for gene-set enrichment analyses can obscure interpretation with strong associations potentially driven by similarities between a given gene and the first principal component of gene expression, regardless of the fMRI data. As such, an adequate number of samples is required to provide stable estimates of PLS regression models. Whilst this is highly specific to the data and research question, Helmer and colleagues make more specific recommendations based on dataset properties (Helmer et al., 2021). Another option to examine multiple receptor systems together is principal component analysis (PCA), within which a set of variables (here, molecular systems from PET/AHBA) is reduced down to a smaller set of components which explain the majority of the variance of the original variables. As with PLS, this allows for large scale examination of multiple molecular mechanisms together, but at the cost of lost interpretability of the results.

### 3.2 Novel molecular perspectives on the functional architecture of the human brain

The human brain is exquisitely complex, with billions of neurones interacting through trillions of shared connections. Understanding this complexity necessitates a wide range of tools to provide distinct but interrelated perspectives. For example, a substantial body of work has examined how the structural connectome shapes the network dynamics observed with fMRI (Miŝic et al., 2016, Seguin et al., 2020, Shen et al., 2015, Suárez et al., 2020, Zimmermann et al., 2016). Analogously, these network dynamics are increasingly appreciated through the lens of their underlying molecular substrates which modulate the activity across this structural connectome. The human brain has been argued to comprise a rich club of densely inter-connected hubs, offering short paths for topological integration between modules (van den Heuvel and Sporns, 2011). However, these hubs come at a significant wiring and metabolic cost; it has been theorised that evolutionary pressures have struck a balance between this cost and the maximisation of integrative capacity (Bullmore and Sporns, 2012, van den Heuvel and Sporns, 2011). In support of this overarching organisational theory, regions of superior and lateral cortex with a high inter-modular degree and long connection distance have transcriptional profiles enriched for oxidative metabolism and mitochondria (Vértes et al., 2016). Interestingly, many of these areas of association cortex that demonstrate long-range cortico-cortical connectivity are also enriched with genes uniquely or disproportionately expressed in supragranular cortical layers in humans, but not in rodents (Krienen et al., 2016). Network strength has also been more broadly correlated to the expression of a set of genes from the AHBA which is largely linked to synaptic function, especially ion channels (Cioli et al., 2014, Richiardi et al., 2015). There is a strong correlation between the expression of the neuronal MCT2 lactate transporter and the function and structure of the brain, offering new evidence for the astrocyte-neuron lactate shuttle hypothesis of neuronal energy supply (Medel et al., 2022). Furthermore, unsupervised learning applied to the AHBA gene expression data and meta-analytic patterns of activity associated with multiple domains of cognition from Neurosynth has demonstrated a ventromedial–dorsolateral gradient of gene assemblies that separates gene sets associated with affective and perceptual functions (Hansen et al., 2021b). Such findings also extend to molecular systems measured using PET. Patterns of receptor/transporter density show greater similarity within intrinsic networks than between them, and brain regions that are more functionally connected also demonstrate similar receptor patterns (Hansen et al., 2022a). Additionally, including information as to the distribution of receptors improves predictions of FC from the structural connectome, highlighting the importance of considering the interrelationships of such systems in generating the network dynamics seen in fMRI. Collectively, these results demonstrate the utility of these approaches to span organisational hierarchies and provide new perspectives on overarching theories of brain organisation. Future work examining the intermediate meso-scale organisation of the brain, in the form of different cell types which are associated with distinct molecular profiles, may help shed further light on how these cellular and molecular building blocks yield the complex network architecture observed in fMRI data.

### 3.3 Molecular systems exert neuromodulatory control over network dynamics associated with cognition

The BOLD signal fluctuations within anatomically distinct cortical and subcortical regions exhibit spatiotemporal relationships with each other, constituting large-scale patterns of correlated and anticorrelated activity (Fox and Raichle, 2007; Ji et al., 2019). Whilst these networks rely on a backbone of structural connections, they undergo dynamic shifts to support the diverse repertoire of brain states required to engage with an ever-changing set of demands (Bassett et al., 2011, Bressler and Menon, 2010, Cole et al., 2021, Cole et al., 2013, Gonzalez-Castillo and Bandettini, 2018, Meehan and Bressler, 2012). These dynamic functional changes have been increasingly studied through the lens of modulatory neurotransmitter systems, which project to overlapping but also distinct brain regions to provide local and global influence over these networks (Shine et al., 2019). Integrating information as to the spatial distribution of these modulatory system’s receptors and transporters offers a new lens through which to examine how the micro-scale modulation of neuronal activity shifts network dynamics in response to ongoing demands. Shine and colleagues explored this systematically, using PCA to derive a smaller set of spatially orthogonal principal components that explained most of the variance in the BOLD signal across seven cognitive tasks (Shine et al., 2019). They then examined relationships between these components, which related to different brain networks as well as aspects of cognition, and the patterns of gene expression for receptors that produce facilitatory (D_1_, ɑ_2a_, M_1_, and 5HT_2a_) and inhibitory (D_2_, ɑ_1a_, and 5HT_1a_) neuromodulatory influence on cognition. These groups of receptors showed differential relationships with the first two principal components: the first was broadly engaged across all tasks and showed positive and negative relationships with the faciliatory and inhibitory groups respectively, whilst the second, which was most strongly linked to social and language processing, showed positive relationships with the catecholamine (dopaminergic and noradrenergic) receptors and negative relationships with serotonergic and cholinergic receptors. Altogether, this highlights the nuance within neuromodulatory control of cognition; whilst there seem to be some domain-general network mechanisms engaged across tasks, other systems engage with greater specificity and these each associate with different subsets of receptor systems. Moreover, how these receptor mechanisms interact remains largely unclear at a systems level, with the methodologies discussed in subsequent sections offering additional tools to explore these complex interrelationships.

### 3.4 The pharmacodynamic effect of drugs are partially explicable through the distribution of their primary targets

Despite almost all commonly used drugs having a well-known pattern of affinity and (ant)agonist activity at a set of target receptors, the relative contribution of each of these receptor systems to the resultant large scale pharmacodynamic response of the brain remains challenging to disentangle. For example, the affinity of different ligands for receptor sub-systems can be unintuitive with noradrenaline able to act on dopamine D1 and D4 receptors under some circumstances (Kobayashi et al., 2022, Root et al., 2015). The molecular-informed fMRI analyses described in this section are well suited to overcome this longstanding challenge. An early and important proof-of-concept study linked spatial correlation of regional cerebral blood flow (rCBF) responses, measured with arterial spin labelling (ASL) fMRI, to PET receptor densities (Dukart et al., 2018), with all seven compounds investigated demonstrating correlations consistent with their known mechanism of action across glutamatergic (AMPA, NMDA, and Kainate), for GABAergic (GABA-A), cholinergic (M1, M2, α4, and β2), noradrenergic (α1 and α2), serotoninergic (5-HT_1a_ and 5-HT_2_), and dopaminergic (D1 and D2) receptor systems. Similar studies have followed. For example, changes in rCBF induced by three antipsychotic drugs were shown to be significantly correlated with D2 receptor density and DRD2 gene expression (Selvaggi et al., 2019). The field has also moved beyond these small-scale investigations. For example, a recent meta-analysis demonstrated that the pooled estimate of the effect of delta-9-tetrahydrocannabinol (THC) across 22 datasets correlated with gene expression of the CB1, but not the CB2 receptor (Gunasekera et al., 2022). Crucially, the functional imaging data included BOLD and ASL fMRI, as well as PET, spanning tasks ranging from reward to sensory processing. As such, the pattern of activation identified related to a set of brain regions engaged under diverse conditions and measured in multiple ways, demonstrating the robustness of this molecular link through the known pharmacology of THC. Future meta-analyses incorporating molecular information may prove a particularly fruitful avenue to delineate robust molecular-functional relationships. However, interpretation of such findings still requires careful consideration given that CB1 receptors are also expressed on astrocytes (Navarrete and Araque, 2008). Similarly, glial cells in general express a wide variety of other neuromodulatory receptors, such as for the catecholamines (Oe et al., 2020). Future work teasing apart the contribution of different cell types will be a crucial next step to map these mechanisms across different scales, with cellular markers such as glial fibrillary acidic protein (GFAP) offering additional complementary meso-scale insights.

Taking an opposing but analogous approach to large-scale molecular informed investigation of psychopharmacology, Luppi and colleagues endeavoured to map the effects of a wide array of psychoactive compounds onto the multi-receptor landscape of the brain (Luppi et al., 2022a), including the following 9 neurotransmitter and neuromodulatory systems: dopamine (D1, D2, DAT), norepinephrine (NET), serotonin (5-HT_1a_, 5-HT_1b_, 5-HT_2a_, 5-HT_4_, 5-HT_6_, 5-HTT), acetylcholine (α4β, M1, VAChT), glutamate (mGluR5, NMDA), GABA (GABA-A), histamine (H3), cannabinoid (CB1), and opioid (MOR). As would be expected, regions with similar chemoarchitecture demonstrated similar changes in FC across different pharmacological challenges spanning psychedelics, anaesthetics, and cognitive enhancers. In a subsequent data-driven PLS regression analysis, psychedelics, and anaesthetics were found to relate to divergent transmitter systems. Moreover, despite these differences, the effects of all drugs mapped onto several hierarchical gradients across multiple domains of anatomy and physiology. These findings highlight the potential key relevance of high CBF, connectivity, and receptor density of parts of transmodal association cortex in mediating the powerful effects of these compounds on cognition and subjective experience. Finally, brain regions which demonstrated susceptibility to pharmacological manipulation also related to patterns of structural pathology associated with a range of neuropsychiatric disorders, including 22q11.2 deletion syndrome, attention-deficit/hyperactivity disorder, autism spectrum disorder, idiopathic generalized epilepsy, right temporal lobe epilepsy, left temporal lobe epilepsy, depression, obsessive-compulsive disorder, schizophrenia, bipolar disorder, and Parkinson’s disease. This study constitutes a significant extension of the basic spatial correlational analyses that preceded it and offers a new perspective on the longstanding challenge of understanding how multiple receptor systems interact to mediate the profound and diverse effects of psychoactive substances on various facets of consciousness and cognition.

The molecular targets of pharmacological interventions can also offer cellular-level insights. For example, propofol is known to act primarily through GABA-A receptors in the mammalian brain (Brown et al., 2010; Trapani et al., 2012). However, there are a host of different cortical GABAergic interneuron subtypes, some of which propofol may preferentially modulate. Craig and colleagues demonstrated that regions in which functional connectivity was significantly reduced under propofol anaesthesia also showed high expression levels of genes that mark the presence of parvalbumin interneurons (Craig et al., 2021). In doing so, these data linked known molecular information from the basic pharmacology of a compound to insights at both the cellular and systems level, resulting in mechanistic insights that span the principal hierarchies of brain function. In the longer term, the vision is that drug actions may no longer be only characterised by their receptor level occupancy, but also potentially by the cell types that express these receptors and mediate the subsequent cellular level effects downstream. Whilst concrete demonstrations of this are lacking, these techniques may prove an important additional tool in drug discovery as well as targeted prescription within clinical settings. As a general note, while studies attempting to link distinct cell types to neuroimaging phenotypes based on the spatial distribution of their transcriptional profiles have been increasingly common, substantial methodological challenges remain. For instance, these analyses necessitate knowledge of the transcriptional profile of each cell category in the human brain, for which there remains no consensus. Thus far, transcriptional profiles have been defined based on a handful of studies sampling tissue from different regions of the post-mortem human brain. However, as single cell transcriptomics studies grow, it is becoming clear that even specific cell types may show differences in transcription within regions. As high throughput transcriptomic techniques continue to characterise distinct neuronal cell types (Zeng and Sanes, 2017) better resources will hopefully become available and allow for more reliable and in-depth cellular decoding of neuroimaging phenotypes.

### 3.5 Molecular information links pathophysiology through to network changes in neuropsychiatric disorders

A key challenge in neuropsychiatric disorders is the multiplicity of implicated mechanisms. As such, linking mechanisms with theorised causal roles in driving symptomatology through to specific measurable perturbations of brain function would be hugely valuable. For instance, several neurodegenerative disorders are associated with hyperphosphorylation, misfolding, and aggregation of the tau protein, with variations in the gene encoding it (MAPT) conferring susceptibility to regional brain dysfunction (Goris et al., 2007, Simón-Sánchez et al., 2009). Rittman and colleagues exploited the known distribution of MAPT from the AHBA to examine the relationships between regional tau protein and network level dysfunction in progressive supranuclear palsy (PSP), hypothesising that higher expression would confer greater susceptibility (Rittman et al., 2016). Indeed, regions with high MAPT expression also demonstrated a strong level of connectivity (hubs), which predicted network perturbation within PSP patients as well as those with Parkinson's disease. Furthermore, executive cognition was impaired in proportion to diminished hub connectivity. Moreover, these findings were not mirrored with another gene (SNCA) which encodes for an additional protein implicated in neurodegenerative pathophysiology. In doing so, they link genetic information through to network function as well as dysfunction and concomitant impacts on cognition within neurodegenerative disorders, showcasing the utility of molecular-enriched analyses to bridge across levels of analysis and further our understanding of how neuropathology might lead to system-level dysfunction.

Ji and colleagues took an alternative approach and examined a heterogeneous cohort of patients with psychosis-spectrum disorders (PSD) with the aim of mapping cognition and psychopathology to imaging data trans-nosologically (Ji et al., 2021). In their initial analyses, they found that the clinical measures could be distilled down to five key components using principal components analysis and that these demonstrated substantially stronger relationships to global brain connectivity (GCB; a measure of FC) than the conventional psychometric measures. Then, using transcriptomic data from the AHBA, they mapped these brain-behaviour relationships onto select molecular systems known to be impacted in the pathogenesis of PSD, including serotonergic and GABAergic receptor subunits as well as markers for interneurons. For example, the map relating to the third principal component strongly correlated with the HTR1E gene, encoding the 5-HT_1e_ receptor, for which there are currently no available ligands. Data-driven decomposition methods applied to symptomatology trans-nosologically and systematically mapped onto underlying molecular systems may constitute a high throughput means by which to facilitate the move towards biologically-informed targeted treatments.

Recently, we have taken a conceptually similar approach to investigate the role of immunomodulatory drugs in mood and fatigue (Martins et al., 2022a, Martins et al., 2022b). Inflammation has attracted considerable attention in recent years as a possible new mechanism contributing to neuropsychiatric disorders in at least some patients. How exactly increases in peripheral inflammation interact with the brain and its inflammatory machinery and might lead to circuit dysfunction is nevertheless under dispute. We used resting state fMRI data from two cohorts of patients treated with interferon-alpha (a pro-inflammatory drug) or anti-TNF-alpha drugs (an anti-inflammatory drug) to map regional changes in response to both treatments and investigate whether these regional changes show spatial concordance with regional distribution of the molecular and cellular neuroinflammatory machinery. We demonstrated links between the pattern of brain changes after interferon-alpha and the expression of genes involved in glial neuroinflammation. Importantly, we showed that it was possible to predict depressive symptoms four weeks after treatment initiation, by using a summary score that quantifies the spatial alignment between each patient’s brain map of treatment changes in connectivity and the canonical signature of neuroinflammatory genes in the brain. This work provides an important example of how integrating molecular information in analyses of neuroimaging data might help to build better mechanistic models of the action of complex drugs, such as immunomodulatory therapies.

### 3.6 Methodical considerations and limitations

The principal challenge associated with the use of correlation analyses to probe associations between brain maps (i.e. fMRI statistical map and PET or gene expression maps) is the inherent spatial autocorrelation that dominates these data, in which functional connectivity, receptor density, and gene expression are more strongly correlated within contiguous brain regions than with more anatomically distant regions (Botvinik-Nezer et al., 2020, Burt et al., 2020, Eklund et al., 2016, Fulcher et al., 2021, Roberts et al., 2016, Shinn et al., 2022, Song et al., 2014, Váša and Mišić, 2022, Wiedermann et al., 2016). This can artificially inflate *p-*values, as two significantly autocorrelated spatial maps are more likely to show strong spatial correlation than maps with random values (Fulcher et al., 2021, Markello and Misic, 2021). The ubiquity of spatial autocorrelation in brain data requires that any study making claims regarding correlations between molecular and fMRI data must demonstrate that this is not solely attributable to lower-order organisational principles. Whilst in-depth discussion of all possible methodological approaches to this problem goes beyond the scope of the manuscript (see in-depth overviews by (Fulcher et al., 2021; Markello and Misic, 2021; Váša and Mišić, 2022)), we briefly describe some of the options available. A common approach has been to try and remove the effect of physical distance through regression (Arnatkevic̆iūtė et al., 2019, Ji et al., 2014). Another is the spin method, which uses the randomised rotation of spherical representations of the cortical surface to randomise anatomical alignment between fMRI and molecular distributions, resulting in a null model that accounts for SA (Alexander-Bloch et al., 2018). However, this presents the obvious limitation of only being applicable to cortical surface maps. A more generalisable method is to generatively model surrogate maps which preserve spatial autocorrelation by matching it to the level present in the input data (Burt et al., 2020, Burt et al., 2018, Vos de Wael et al., 2020). Widespread implementation of these approaches will be crucial to identifying meaningful multimodal relationships, and the development of novel statistical methods must keep pace with advances in molecular-enriched analyses in order to maintain statistical rigour.

## 4 Molecular-enriched network analyses

Despite offering initial insights through ease of use, the spatial correlation analyses described above are limited in their ability to exploit the rich spatiotemporal dynamics of brain-wide BOLD fluctuations. In order to overcome this, novel analytical approaches have been developed to enrich fMRI data with the molecular information provided by PET and SPECT imaging (Cercignani et al., 2021, Dipasquale et al., 2020, Dipasquale et al., 2019, Lawn et al., 2023, Lawn et al., 2022; Daniel Martins et al., 2022a, Martins et al., 2022b; Wong et al., 2022), as well as gene expression data from the AHBA (Salvan et al., 2022). Compared to the spatial correlation analyses, the derivation of molecular-enriched networks offers two key benefits. Firstly, it exploits both the spatial and temporal domains of the BOLD signal, providing more nuanced insights into the neurobiology. Secondly, it allows for network-based fMRI analyses that provide additional information regarding receptor specificity while also spatially locating regions of differential FC, allowing for conceptual links to prior molecular, neuroanatomical, and neurocognitive work. In short, molecular-enriched functional networks show the analytic flexibility of conventional network analyses but more directly engage with the underlying neurobiology. Thus, despite similarities with spatial correlation analyses, molecular-enriched networks allow for targeted and hypothesis-driven delineation of pharmacological effects and disease states, offering opportunities to link these through novel diagnostic and predictive biomarkers.

### 4.1 Methodology

**Fig. 2:**
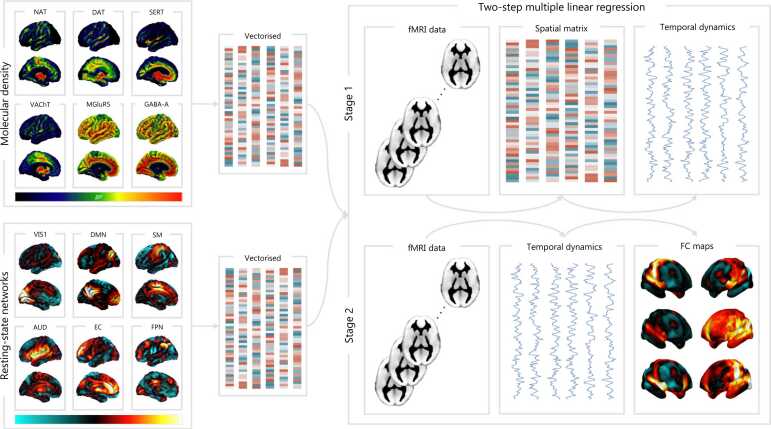
**: An overview of the dual regression approach utilised within conventional RSN and REACT analyses.** Example templates of molecular systems from PET are shown in the top left, analogous to the RSN templates derived from independent components analyses shown in the bottom left. These can be vectorised and entered into the same two-step multiple linear regression framework as shown on the right. In the example of REACT, during stage 1 PET receptor density information is used as a spatial matrix and regressed against the whole brain fMRI data to generate the temporal dynamics associated with each molecular system. In stage 2, these temporal dynamics are then regressed against the BOLD time series in each voxel to generate molecular-enriched FC maps. Finally, these maps can be compared across different experimental conditions such as drug vs placebo. NAT; noradrenaline transporter. DAT; dopamine transporter. SERT; serotonin transporter. VAChT; vesicular acetylcholine transporter. MGluR5; metabotropic glutamate receptor 5. VIS1; medial visual network. DMN; default mode network. SM; somatomotor network. AUD; auditory network. EC; executive control network. FPN; frontoparietal network.

This employs the same two-step multiple linear regression approach used within conventional resting state network (RSN) analyses to estimate subject-specific RSNs from group-level probabilistic maps derived within independent components analysis (Nickerson et al., 2017). However, instead of conventional RSN templates, these novel methods project the fMRI data onto the space of specific neurotransmitters derived from PET and SPECT imaging, or from the AHBA, to derive biologically informed networks of functional connectivity (Fig. 2). This approach was named REACT by Dipasquale and colleagues the first time it was defined and applied in a pharmacological dataset (https://github.com/ottaviadipasquale/react-fmri (Dipasquale et al., 2019; Dipasquale and Frigo, 2021)). REACT typically uses in vivo templates of the distribution density of receptors and transporters, estimated by averaging PET and SPECT data of datasets of healthy controls, as a set of spatial regressors in a first general linear model (GLM) against single-subject fMRI data to extract the dominant BOLD fluctuations associated with the distribution of those molecular systems. In this step, regions with higher molecular density contribute more to the estimation of the BOLD fluctuations related to the molecular system than those with lower density. The output of the first GLM is then used in a second GLM against the single-subject fMRI data to estimate subject-specific molecular-enriched functional maps for each molecular system investigated. REACT-derived molecular-enriched networks can be thought of as delineating regions whose time series are functionally coupled (i.e., positively correlated) or un-coupled (i.e., negatively correlated) to the BOLD fluctuations within ‘core’ regions for a certain transmitter system, i.e., regions highly enriched with that neurotransmitter. The resulting molecular-enriched networks can be compared between groups (e.g., patients vs controls), conditions (e.g., rest vs naturalistic stimuli), or states (e.g., drug vs placebo) as well as correlated with variables of interest (eg: clinical, psychometric, or pharmacokinetic measures). This approach integrating molecular information into fMRI networks has been recently extended to gene expression data and used to examine receptor-enriched networks in both mice and humans using gene expression data from the Allen Mouse/Human Brain atlases to derive so-called “serotonin receptor networks (SRNs)” (Salvan et al., 2022).

The vast majority of molecular-enriched fMRI analyses to date have focussed upon resting state data, largely due to its relative simplicity, abundance, and possibility to explore across a range of basic neural mechanisms u without being restricted to a specific cognitive phenomenon. However, there are limits to which insights can be gleaned from rest alone, with dynamic neural activity associated with task-based data seemingly better placed to capture behaviourally relevant variability (Finn, 2021). Moreover, some molecular systems are differentially engaged under different conditions and brain states. For example, noradrenergic modulation seems to produce largely opposite effects on network dynamics during task and rest (Coull et al., 1999, Pfeffer et al., 2021, Shine et al., 2018). As such, studies investigating noradrenergic circuits solely at rest will likely miss fundamental insights into the mechanisms through which they shape network dynamics and cognition. This interplay between brain state and pharmacological manipulation is highly unlikely to be limited to noradrenaline. REACT offers two additional options to assess task-based changes in molecular-enriched connectivity. Firstly, a modification of the generalised psycho-physiological interaction analysis (gPPI) can be conducted in which the subject-specific dominant BOLD fluctuation related to a certain molecular system, derived from the first GLM of a REACT analysis, is combined with the task regressor by means of scalar multiplication, to produce an interaction regressor to be used in the second GLM to estimate a molecular-enriched task-dependent network (Wong et al., 2022). When expanding this approach to different molecular systems and tasks, the resulting networks can be used to examine the differential engagement of those systems under different task conditions. Alternatively, the standard analytical REACT pipeline can be used to examine cognitive and perceptual systems using naturalistic stimuli engaging lower-level sensory processes (e.g., audition) as well as higher-level cognition (e.g., processing and comprehension of the auditory content) without necessitating behavioural responses. This allows for REACT to be applied across the entire fMRI time series, which can subsequently be compared to a baseline of true 'resting state’ (Lawn et al., 2023). As a future development step, novel features to explore task data, and in general to explore the dynamical shifts in functional connectivity, will be important to better exploit the rich information contained within fine-grained temporal features (Hutchison et al., 2013). To date, only static measures of connectivity have been utilised within molecular-enriched network analyses, with significant scope to examine how these systems are shaped over time by naturalistic stimuli or an acute pharmacological challenge.

### 4.2 Molecular-enriched networks can help disentangle receptors’ contribution to the brain’s pharmacodynamic response

As seen in Section 3.3, many drugs have rich pharmacology with actions on a multitude of receptors, which makes the disentanglement of their relative contribution to the large-scale pharmacodynamic response challenging. Molecular-enriched analyses show promise in bringing new perspectives to these network changes, by mapping them onto putative receptor mechanisms. For example, methylphenidate binds to both the dopamine transporter (DAT) and the norepinephrine transporter (NAT), with a lack of consensus as to the relative importance of these two systems for mediating its actions. Recently, REACT has demonstrated that this drug produces significant effects on connectivity within the key sensorimotor regions of the functional network related to the former, but not the latter, potentially indicating a greater contribution of dopaminergic circuits to its pharmacodynamics (Dipasquale et al., 2020). Similarly, LSD is thought to act primarily through the 5HT_2a_ receptor (Kraehenmann et al., 2017, Preller et al., 2018, Preller et al., 2017), although it also shows affinity and agonist activity at the 5-HT_1a/b_, 5-HT_6_, 5-HT_7_ as well as D1 and D2 receptors (de Gregorio et al., 2016, Marona-Lewicka et al., 2002, Nichols, 2004, Passie et al., 2008). A recent exploratory study using REACT found that dopaminergic-enriched FC within somato-motor, superior parietal and insular/opercular regions was related to aspects of psychedelic phenomenology including disembodiment and impaired control/cognition induced by LSD (Lawn et al., 2022). Importantly, these did not correlate with the connectivity of serotonergic receptor sub-systems within the same regions. Delineating the receptors through which drugs impart their effects opens many doors within drug development. For example, all pharmacological compounds produce side effects. Through understanding the key molecular targets, compounds which more selectively interact with these can be developed, improving tolerability and expanding the therapeutic window.

### 4.3 Novel diagnostic and predictive neuroimaging biomarkers

There is a substantial ongoing drive to develop novel biomarkers for brain disorders. However, typical fMRI measures such as correlation of BOLD signal between regions remain extremely challenging to translate into clinically meaningful applications, with fMRI-based biomarkers having extremely limited implementation in clinical practice to date. There are a host of interrelated reasons for this (Woo et al., 2017), but one key issue is that conventional fMRI biomarkers remain abstracted from the underlying molecular-level disease mechanisms to which we wish to target interventions. Providing a biological grounding to FC holds significant promise for novel biomarkers and the concomitant development of targeted treatments informed by biological mechanisms. In a recent study, mapping molecular-enriched networks with REACT has provided novel insights into aberrant brain function and pharmacotherapy in the context of social processing in autism (Wong et al., 2022). First, the authors demonstrated that serotonin transporter (SERT) enriched FC was greater during the presentation of emotional faces than shapes (faces>shapes) within a range of regions implicated in facial emotion processing including the superior temporal gyrus, superior parietal lobule, posterior cingulate, amygdala, prefrontal cortex, striatum, and fusiform gyrus. Furthermore, individuals with autism had reduced SERT-enriched faces>shapes responses within the right-ventromedial prefrontal cortex. Moreover, the authors showed that this deficit was partially normalised towards neurotypical levels by citalopram, a selective serotonin reuptake inhibitor (SSRI) acting on the SERT.

In another study, REACT has also provided promising findings in chronic pain (Daniel Martins et al., 2022a, Martins et al., 2022b), which is increasingly understood to be partially driven by, and produce, perturbations in supraspinal functional circuits (Barroso et al., 2021). However, treatment responses are highly heterogeneous, likely due to divergent underlying mechanisms within patients sharing the same diagnoses (Woolf, 2010). Prospective stratification based on psychophysics has shown some promise for the targeted prescription of analgesics (Demant et al., 2014), but novel and effective predictive biomarkers are desperately needed (Tracey et al., 2019). In this REACT-based study, patients suffering from osteoarthritis demonstrated increases in NET- and SERT-enriched FC compared to healthy controls within predominantly frontal regions as well as decreases in SERT-enriched FC in the middle/superior temporal gyrus (Daniel Martins et al., 2022a, Martins et al., 2022b). Moreover, baseline noradrenaline transporter (NAT)- and SERT-enriched FC across diverse regions spanning frontal, parietal, and occipital cortices showed predictive value as to which of these patients would respond to duloxetine, a drug acting primarily on these two transporter systems (Bellingham and Peng, 2010). Additionally, baseline DAT-enriched FC within the right opercular and parietal cortex was predictive of placebo responders, in line with dopaminergic roles in expectancy and placebo mechanisms (de la Fuente-Fernández et al., 2001, Scott et al., 2008, Scott et al., 2007) as well as associations between rCBF in osteoarthritis and dopamine D2 receptor expression (Vamvakas et al., 2022). This work provides preliminary proof-of-concept evidence supporting the idea that molecular-enriched analyses such as REACT might be helpful not only to characterise the dysfunction of molecular-enriched functional networks in disease, but also to sub-stratify patients based on the underlying mechanisms of dysfunction, allowing for targeted prescription of analgesics. Subsequent work within different chronic pain populations and using other analgesic drugs will be crucial to demonstrate the generalisability and scalability of molecular-enriched functional networks as novel biomarkers within chronic pain (Soliman et al., 2023). Additionally, characterising those who may be placebo responders may offer additional opportunities to exploit these effects for therapeutic benefit, as within open-label placebo (Carvalho et al., 2016, Finniss et al., 2010, Kaptchuk and Miller, 2015).

These studies exemplify the potential of molecular-enriched analyses to provide novel insights into the molecular underpinnings of cognition and perturbation in disease as well as providing further links to pharmacology. The application of these methods to neuropsychiatric disorders more broadly may contribute to efforts to finally transcend non-specific accounts of network-level dysfunction and bring fMRI closer to real clinical applications.

### 4.4 Methodical considerations and limitations

There are some important considerations for molecular-enriched network analyses which merit discussion. However, before discussing the more specific limitations of this methodology, it is worth noting that the same challenges that apply to conventional network-based analyses also apply here. For example, psychosocial variables such as education or socioeconomic status can confound between group or regression analyses whilst sources of physiological noise can shape BOLD dynamics, requiring careful data pre-processing (Chang et al., 2009). Furthermore, small sample sizes present a similar challenge within molecular-enriched analyses to conventional fMRI analyses, and crucially these depend on the research question, design of the study, and magnitude of the expected effects. As such, whilst one-size-fits-all recommendations would be unhelpful here, we emphasise the need to consider sample size and power of molecular-network enriched analyses, especially in contexts where samples have been consistently small, such as psychedelic pharmacoimaging.

Firstly, one of the key assumptions behind the use of molecular-enriched networks is that they might capture facets of neurotransmission, which so far has not been rigorously demonstrated. In humans, testing this working model has been mostly restricted to pharmacological interventions targeting specific neurotransmitter systems. Here, plausible links between drug targets and target-enriched FC have been demonstrated (Dipasquale et al., 2020, Dipasquale et al., 2019, Lawn et al., 2023, Lawn et al., 2022; Daniel Martins et al., 2022a, Martins et al., 2022b; Wong et al., 2022), strengthening the confidence that molecular-enriched networks are sensitive to variations in concomitant neurotransmission. However, the complex patterns of affinity of most drugs and the risk of non-specific vascular effects that might affect neuroimaging signals necessitate further confirmatory studies. One unexplored avenue is the use of blocking studies, within which the effects of a drug on molecular networks can be examined with and without an antagonist to the primary target, providing a causal manipulation of the system of interest which should be reflected in the molecular-enriched functional networks. Another option would be to utilise manipulations of molecular systems within animal models, as recently done by Salvan and colleagues (Salvan et al., 2022) to show that pharmacological and optogenetic manipulation of the serotonergic system modulates the SRNs in a receptor sub-system-specific manner. A similar study has also recently shown that the serotonin receptor gene expression patterns explain a large proportion of the variance in the BOLD signal following optogenetic stimulation of the dorsal raphe (Hamada et al., 2022). Future methodological work expanding the scope of this approach beyond serotonin will be an important step forward to inspect construct validity across neurotransmitter systems. Moreover, with multimodal data acquisition protocols gaining traction, future studies investigating how inter-individual differences in molecular-enriched network connectivity relate to those in neurochemistry (i.e., as assessed with PET/SPECT or magnetic resonance spectroscopy) could constitute an interesting way forward. This may also offer opportunities to explore the varying timescales over which different measures fluctuate such as simultaneously acquired PET and fMRI data (see (Cecchin et al., 2017) for a detailed review), offering additional opportunities to expand these analyses beyond static connectivity and explore time-varying molecular-functional relationships.

Secondly, the choice of receptor systems employed requires careful consideration. Broadly speaking, two approaches have been taken. Studies examining drug effects generally select a priori receptor systems for which the compound has known affinity and (ant)agonist activity (Dipasquale et al., 2020, Dipasquale et al., 2019, Lawn et al., 2023, Wong et al., 2022). However, in other circumstances where there is generally a less clear rationale for highly specific receptor sub-system involvement, e.g., when exploring the brain mechanisms underlying a certain disorder or examining differences in the reconfiguration of receptor-enriched networks between task and rest, the most common approach has been to use the spatial distribution of the transporters that are engaged in the movement of neurotransmitters across synaptic and vesicular membranes, which provide a coarse grain marker for the influence of a given neurotransmitter system over a given brain region (Cercignani et al., 2021, Lawn et al., 2023; Daniel Martins et al., 2022a, Martins et al., 2022b). Follow-up analysis employing specific receptor subsystems can then be used to probe significant findings associated with a transporter-related network. This is especially effective when investigating the modulatory neurotransmitters (such as the monoamines and acetylcholine) whose widespread arborisation from small brainstem, midbrain, and forebrain nuclei produces a spatiotemporal influence over the BOLD signal that lends itself well to REACT. For example, if a study were to find differences in connectivity enriched with the DAT, a follow-up analysis could employ the distributions of the dopamine D1 and D2 receptors to attempt to gain further insights into the receptor specificity of these findings.

Thirdly, the applicability of PET and AHBA data derived in normative cohorts to those with substantial differences remains contentious. Many molecular systems, such as noradrenergic modulatory projections from the locus coeruleus (Betts et al., 2017, Clewett et al., 2016, Hämmerer et al., 2018, Lee et al., 2018, Liu et al., 2020, Liu et al., 2019, Manaye et al., 1995, Porat et al., 2022, Shibata et al., 2006), change as a function of age. No studies to date have examined the significance of such differences on the derivation of molecular-enriched networks within older cohorts, despite this wide ranging and robust evidence for age-related noradrenergic changes. Direct comparison of PET maps matched to demographics for a given cohort to those conventionally used may be of benefit, although hindered by data availability. Similarly, neuropathology can and does affect different neurotransmitter systems, calling into question the use of distributions from normative samples. For example, in Parkinson’s disease (PD) patients showing dopaminergic denervation (Lotharius and Brundin, 2002), one would expect to find network changes related to the spatial distribution of the dopaminergic receptors. However, it is important to point out that as with classic RSN analysis, where network templates obtained from an independent dataset of healthy controls are used to estimate the subject-specific RSNs of healthy and pathological datasets, the templates used in molecular-enriched analyses have the principal purpose of weighting the BOLD signal according to the importance of each region in a specific molecular system. Although the use of molecular templates of healthy subjects on clinical populations could be seen as a sub-optimal strategy, the use of patient-based templates is associated with at least two major disadvantages. First, if the molecular template is estimated from data belonging to a separate cohort of patients, it is likely that the molecular alteration defined in the template will only partly overlap with the dataset under exam due to factors that might differentially impact the molecular substrate (e.g., different stages of pathology, mechanistic heterogeneity within diagnostic categories, comorbidity, etc). Second, the functional networks estimated from such templates might not properly weight the contribution of the core regions of the molecular systems examined. This might result in networks that do not fully capture the features of the molecular systems.

Finally, there is a certain degree of spatial overlap between different molecular systems (Beliveau et al., 2017, Dipasquale et al., 2019, Lawn et al., 2023, Lawn et al., 2022). To account for this, it is important to include all molecular systems within the same model, instead of running separate models for each system, which would lead to an omitted-variable bias. This bias occurs when a statistical model (here, multiple linear regression) omits an independent variable (a molecular system) that is both a determinant of the dependent variable (the BOLD signal) and correlated with one or more of the included independent variables. This yields an unpredictable attribution of the effects of the missing variables to those variables that are included. However, as noted above in relation to PLS regression (3.1), doing so does not solve the collinearity issue. One potential path forward may be to derive components from the different receptor system maps, in order to identify low dimensional molecular representations which separate out shared and independent variance. However, given the associated challenges of interpretability, using dimensionality reduction approaches might be only an attractive alternative when the model presents severe multicollinearity, while for cases of moderate correlation between molecular templates, it would be acceptable to include them in their original form. Variance inflation factors (VIF) provide a measure of multicollinearity within a regression model, quantifying the correlation between predictor variables, as done by Lawn and colleagues to test the collinearity of five molecular systems (Lawn et al., 2023). Typically, VIFs higher than 1 and up to 5 suggest that there is a moderate correlation, but it is not severe enough to warrant corrective measures, while values greater than 5 represent critical levels of multicollinearity where the coefficients are poorly estimated and alternative analytic approaches, such as the dimensionality reduction mentioned above, are hence required (García et al., 2014, Kim, 2019, Kovács et al., 2005, O’Brien, 2007).

## 5 Computational modelling approaches to link molecular and functional systems

Advances in computational neuroscience have increasingly allowed for the modelling of whole-brain dynamics in silico (Breakspear, 2017), offering novel opportunities to explore how different receptor systems contribute to dynamic brain activity. Whilst the methods described thus far attempt to reason from systems-level dynamics down towards molecular mechanisms, whole-brain computational models attempt to build up from the fundamentals of neuronal microcircuitry to simulate systems level dynamics (Breakspear, 2017, Cofré et al., 2020, Stinson and Sullivan, 2017). As with the spatial correlation analyses in Section 3, this moves away from simply localising brain regions associated with a cognitive or clinical mechanism of interest and towards a trans-hierarchical account of how complex systems interact across scales and regions. Compared to the non-generative approaches described in previous sections, this comes with two important strengths. First, it offers a platform to easily test multiple competing hypotheses about specific changes in microcircuitry and how they could lead to macro-scale changes by fitting and comparing different models. Second, rather than relying on post hoc interpretations, using generative models requires formalizing hypotheses as to possible mechanisms a priori, improving precision, rigour, and transparency.

### 5.1 Methodology

**Fig. 3:**
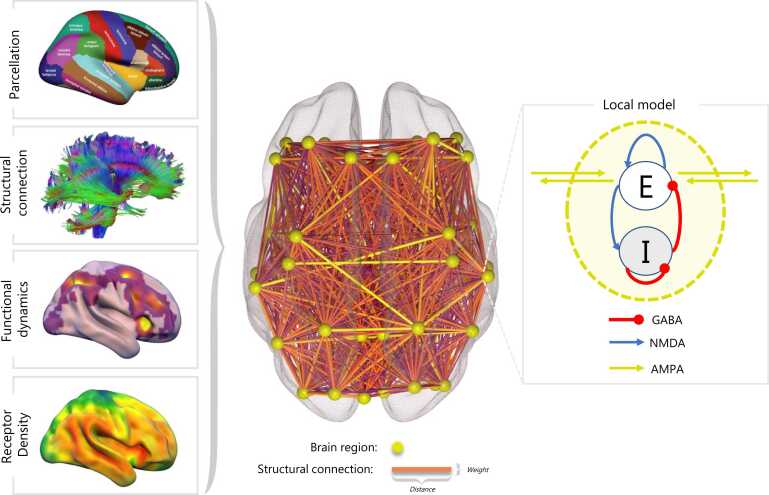
An overview of the necessary multimodal constituents for a whole brain computation model. A parcellation defines a series of nodes (brain regions) which are connected based upon structural connectivity from diffusion tensor imaging (DTI). Each node is modelled through biophysically plausible excitatory (E; NMDA) and inhibitory (I; GABA) dynamics as shown in the local model on the right. In addition, molecular density information from PET can be incorporated into the model to shape specific aspects of the model such as neuronal gain.

The full details of generating whole brain models are beyond the scope of this review, though Cofre and colleagues provide a detailed overview of the key methodological aspects (Cofré et al., 2020). In short, they require a parcellation of the brain to delineate the different regions for which local dynamics and global interactions will be modelled, an anatomical connectivity matrix that provides an approximation of the white-matter fibre connectivity between these regions, and a model of local dynamics for which a vast array of different options have been proposed (Breakspear, 2017, Cofré et al., 2020)(Fig. 3). Common choices for local dynamics include neural mass and mean field models, i.e., a series of differential equations constituting an abstracted yet realistic model of neuronal interactions that can approximate the observed emergent macroscale dynamics in experimental data (Breakspear, 2017, Cofré et al., 2020). As such, causal manipulation of the constituent components (parameters of the model) of these models allows for systematic examination of the counterfactual consequences of modifying different facets of brain function (the biological mechanisms relating to those parameters).

In the context of molecular-enriched analyses, PET receptor density information can be incorporated into these models (Deco et al., 2018, Jancke et al., 2021, Luppi et al., 2022b) to explore their capacity to model experimental data under conditions in which that receptor system is putatively engaged. However, these biological mechanisms of interest must be integrable into specific model parameters. This is particularly tractable in the context of pharmacological manipulation of neuromodulatory systems, in which the effects of a drug can be modelled by including the scaling of neural gain according to the relative distribution of a receptor system to try to capture the change in dynamics observed experimentally. This provides a powerful means by which to test explicit hypotheses regarding the role a given receptor plays in, for example, producing the network changes seen under the effects of a drug that putatively binds to it. As such, these sources of molecular information from PET or the AHBA can be thought of as a source of heterogeneity across the whole brain model, with the opportunity to shape many additional parameters such as the time constant tau, resting membrane potential, action potential threshold, and maximum neural firing rate (Bomkamp et al., 2019, Demirtaş et al., 2019).

### 5.2 Modelling the psychedelic state

To date, whole brain models that incorporate molecular information have largely been applied in the context of pharmacological challenges, often with psychedelic compounds. This has primarily been undertaken with an emphasis on demonstrating the utility of these methods as well as advancing the biophysical complexity. In an early example of this, Deco and colleagues explored the pharmacodynamics of LSD (Deco et al., 2018). First, they generated a dynamical-mean field model which tried to approximate FC during a placebo condition. They then included 5-HT_2a_ receptor density information from PET to modulate neural gain by selectively altering the recursive excitatory and inhibitory local microcircuits of different brain regions based on its local density. The logic of this approach is analogous to the aforementioned spatial correlation and molecular-enriched network analyses; the pharmacodynamic effects of the drug are at least partially explicable by the distribution of its primary targets. Incorporating the 5-HT_2a_ receptor distribution produced a better fit between the simulated and experimental data under the LSD condition than the other serotonergic receptors (5-HT_1a_, 5-HT_1b_, 5-HT_4_, and 5-HTT). However, the 5-HT_1a_ receptor model performed significantly better than the others and nearly as well as that using 5-HT_2a_, highlighting the need to consider the effects of multiple receptor systems.

Burt and colleagues generated a similar model using AHBA transcriptomic data to simulate the effects of LSD on global brain connectivity, but also examined the differential contribution of excitatory and inhibitory microcircuitry as well as attempted to fit the model at a subject-specific level (Burt et al., 2021). Their results were most parsimonious with LSD producing an increase in excitation-inhibition ratio mediated by 5-HT_2a_ modulation of cortical pyramidal neurones, although many of the other serotonergic and dopaminergic targets also resulted in moderate concordance between simulated and experimental data. Another interesting approach diverges from whole brain models but applies the same fundamental logic to network control analysis (Parker Singleton et al., 2022). Specifically, the authors examined the energy landscape of the brain and quantified the energy required to transition between recurrent network states during the psychedelic state and placebo, which features prominently in the REBUS model of psychedelic action (Carhart-Harris and Friston, 2019). Akin to the whole brain modelling, incorporating the 5-HT_2a_ receptor molecular density from PET reduced the resulting transition energies, mirroring those within the psychedelic condition (Parker Singleton et al., 2022). Similarly, whilst the 5-HT_2a_ was the most effective at reducing transition energies, the 5-HT_1a/1b_ receptors also produced a substantially greater effect than a spatial uniform control map. Collectively, these studies demonstrate the power of these approaches to examine the specific contributions of receptor sub-systems to the large-scale pharmacodynamic response of the brain to psychedelic compounds. However, whilst they collectively allude to the key role of the 5-HT_2a_ receptor, they also highlight the pharmacological complexity interactions across organisational hierarchies as well as the likely relevance of other receptor systems which also show reasonable goodness of fit.

In a significant advance upon these initial papers modulating gain based on the distribution of the 5-HT_2a_ receptor, Kringelbach and colleagues developed a mutually coupled neuronal-neurotransmitter whole brain model which attempts to better grapple with the biophysical complexity of neuromodulatory control over network dynamics during the administration of psilocybin (Kringelbach et al., 2020). A standard whole-brain model was created to simulate the placebo condition. However, to model the slow serotonergic metabotropic receptor-mediated effects of the drug, they employed a separate set of differential equations which characterise release and re-uptake dynamics coupled to the standard pools of excitatory and inhibitory neurones in each brain region through the structural connectivity of the raphe nucleus to the rest of the brain (Joshi et al., 2017). The neurotransmitter currents in each region are also scaled by the density of the 5-HT_2a_ from PET. Finally, the reverse coupling is also modelled from the neuronal compartments back to the population by incorporating the raphe nucleus neuronal firing rate into the release and re-uptake equations. Overall, the interaction between these different dynamical systems was found to be fundamental for explaining the empirical data observed during the administration of psilocybin. In systematically removing different aspects of the model (e.g., by removing feedback dynamics or using a reshuffled version of the 5-HT_2a_ receptor map), they demonstrate that each is important for fitting to the empirical data. This more complex recurrent modelling approach provides an exciting opportunity to bring these models towards biological plausibility. A crucial next step will be to explore how multiple interacting neuromodulatory systems work in concert to shape network dynamics under drugs with rich pharmacology, and disease states in which a multiplicity of receptor systems are implicated. One option may be to utilise the novel multiscale dynamic mean-field models with synaptic gating equations linked to neurotransmitter concentrations, but with heterogeneity across regions included as a function of receptor density or gene expression (Naskar et al., 2021).

### 5.3 Beyond psychedelia: using mean field models to understand the effects of propofol on consciousness

A longstanding question in consciousness research is to what extent diverse states of unconsciousness, such as anaesthesia or clinical disorders of consciousness (DOC), result from shared or divergent mechanisms. Utilising a similar framework to the aforementioned psychedelic studies, Luppi and colleagues explored whether the characteristic network changes seen in the anaesthetic state and DOC result from inhibition and connectome perturbation (Luppi et al., 2022b). They first generated a mean-field model fit to experimental data in awake healthy individuals before examining whether scaling the inhibitory local gain in proportion to the GABA-A receptor density derived from PET could improve the fit of the model to experimental data collected in the same individuals under propofol anaesthesia. Specifically, they introduce the ionotropic receptors in the excitatory/inhibitory balance of the population equations. This GABA-A receptor-mediated scaling improved goodness-of-fit between simulated and empirical imaging data in the anaesthetised state, over and above that seen for both a modified randomly permuted “scrambled” or uniform density versions of the receptor density map. This strongly implicates GABA-A-mediated inhibition in the characteristic macroscale dynamics observed during propofol anaesthesia. Fascinatingly, the GABA-A-informed modelling also improved the capacity to model network architecture associated with DOC, suggesting that altered inhibitory tone is associated with aberrations in consciousness more broadly. However, this was seen for both the actual distribution of GABA-A receptors as well as the randomly permuted version, suggesting that whilst propofol anaesthesia is specifically contingent on the regional specificity of altered inhibition, this occurs on a more global level in DOC. Finally, they also sought to examine whether meso-scale knowledge as to the spatial distribution of different inhibitory interneurons (determined using transcriptomic profiles from the AHBA) through which this inhibition might be mediated could allow for regional variability in the inhibitory scaling enacted through the GABA-A receptor. No significant differences were seen in model fit by including this additional information, although this premise holds significant potential, and subsequent work which provides intermediate links through meso-scale organisational principles offers an enticing unexplored avenue for these methods. This cross examination of pharmacology and pathophysiology utilising GABAergic molecular information highlights the potential of these techniques, allowing for a nuanced dissection of similarities and differences between these analogous but distinct brain states hitherto relatively un-interrogable by conventional fMRI techniques.

### 5.4 Network dynamics through the lens of transcriptomic-informed whole-brain models

Beyond the pharmacological studies attempting to model psychopharmacological effects through receptor densities of the drug's primary targets, whole brain models have also incorporated transcriptomic data from the AHBA to study the functional relevance of excitatory and inhibitory receptor expression (Deco et al., 2021). By comparing different micro- and meso-scale organisational properties’ ability to constrain regional heterogeneity and tune the dynamics of each brain region, whole-brain models can begin to delineate their relative importance to recapitulate the complex dynamics in fMRI data. Specifically, the authors examined excitatory and inhibitory (AMPA, NMDA, and GABA) receptor expression, the dominant mode of brain-specific gene expression (the first principal component of 1926 brain-specific genes (Burt et al., 2018)), and T1weighted/T2weighted ratios (a myeloarchitectural proxy for cortical hierarchy (Burt et al., 2018)). Interestingly, the balance of excitatory and inhibitory receptor expressions most faithfully reproduced experimental measures of both static and dynamic FC as well as provided the greatest ignition capacity (i.e., the capacity for a sensory stimulus to trigger self-supporting, transient, metastable, and distributed activity across a network, which is thought to be necessary for it to be consciously experienced (Deco et al., 2021; Dehaene and Changeux, 2011; Mashour et al., 2020)). This represents only the most basic initial application of this approach, but it highlights the utility of these models to explore organisational principles. Specifically, they allow for causal manipulation of constituent components in a manner ethically and practically infeasible in human subjects. In doing so, increasingly complex models may allow for fundamental insights into what micro- and meso-scale mechanisms shape different facets of network dynamics more broadly.

### 5.5 Modelling neuropsychiatric disease states

In the longer term, these models may provide an important theoretical link between the network dysfunction increasingly characterised in clinical populations to the underlying driving cellular and molecular mechanisms to which we can target interventions (Deco and Kringelbach, 2017, Deco and Kringelbach, 2014, Gilson et al., 2020, Jancke et al., 2021, Kringelbach and Deco, 2020). Indeed, different neurotransmitter systems such as dopamine and serotonin have been implicated in shaping selected RSNs (Conio et al., 2019), which in turn have been hypothesised to underlie different neuropsychiatric disorders such as schizophrenia and bipolar disorder, highlighting the need to disentangle these relationships to delineate disorder- and symptom-specific mechanisms. Whilst early work has indicated the utility of modelling disease-related network dysfunction (Cabral et al., 2012, Cabral et al., 2012), the application of molecular-informed whole-brain models to address this question remains relatively unexplored and offers an important avenue for future mechanistic elucidation. One example of such work is using The Virtual Brain (TVB; thevirtualbrain.org) neuroinformatics platform (Ritter et al., 2013, Sanz-Leon et al., 2015) to model the perturbed network dynamics seen in patients with Alzheimer’s disease (Stefanovski et al., 2019). Despite being central to pathology of Alzheimer’s, the causal role of the amyloid beta protein in producing the hallmark symptoms of dementia remain contentious (Tolar et al., 2019). By including spatial information as to the distribution of the amyloid beta protein from PET, Stefanovski and colleagues demonstrated that their whole brain models within which amyloid beta modulates regional excitation-inhibition balance could reproduce previously described slowing of frequencies in local field potentials and simulated electroencephalograms. Interestingly, this was reversible by modelling the effects of the NMDA receptor antagonist memantine. This work highlights the capacity of such models to additionally work with electroencephalography data as well as investigate treatment mechanisms. In doing so, a causal understanding of putative treatments may be examined in a high-throughput way, potentially providing a crucial new tool for therapeutic development.

### 5.6 Methodical considerations and limitations

To date, whole brain models have considered only one receptor system or examined different receptor systems in isolation. Future models incorporating multiple receptors and accounting for their complex non-linear interactions will be important to meaningfully capture the full set of mechanisms through which the effects of drugs and disease are enacted. However, this poses additional computational and inferential challenges, such as overfitting models to noise when applied to empirical data. The existing work has also only utilised parcellated data whose spatial resolution may overlook more fine-grained pharmacodynamic effects. However, recent analytic and numerical advances have already reduced the computational expense of dynamic mean field models, with good concordance between parcellations of 100 and 1000 regions, making these methods more accessible to the broader neuroscientific community (Herzog et al., 2022). Similarly, different parcellations will likely affect model fitting, especially if they do not include subcortical regions or specific key regions such as the claustrum, which is putatively important for the mechanisms of psychedelic drugs (Doss et al., 2022). As with all multimodal analyses, these computational models rely upon high-quality data. For example, the tractography-based connectome generally used in these models is also known to be incomplete, and better connectivity parameters will likely improve future models (Herzog et al., 2020, Markov et al., 2014). Finally, the combined use of bulk transcriptomics and single-cell RNA-sequencing may allow for the application of other methods, such as multicompartment models, which explore the meso-scale contribution of specific cell types to pharmacological mechanisms (Burt et al., 2021, Burt et al., 2018, Lake et al., 2016). This endeavour would benefit from efforts trying to map single-cell gene expression at higher spatial resolutions (i.e., cortical layers), which are starting to emerge yet are still confined to very few regions of the human brain.

## 6 Conclusion

The novel methodologies outlined here bring together micro-scale molecular level information with macro-scale systems dynamics, offering important insights into the trans-hierarchical functional organisation of the brain in both health and disease. Despite offering initial opportunities through ease of use, spatial correlation analyses are increasingly being used for large scale mapping of multiple molecular systems to multivariate fMRI measures. Receptor-enriched network analyses allow for the characterisation of spatiotemporal relationships between the BOLD signal and the distribution of molecular systems, with maps of receptor-enriched connectivity amenable to conventional inter-subject analyses, including voxel-wise regression with behavioural and clinical measures of interest. Finally, whole brain modelling allows for the systematic and causal manipulation of micro- and meso-scale facets of brain structure and function, such as the contribution of different receptor systems, to examine the counterfactual consequences on resultant simulated network dynamics. Moreover, in linking system-level dynamics to their molecular substrates, we can begin to transcend the fragmentation of neuroscience as a field by bringing together findings across multiple levels of analysis. We hope that this may ultimately help catalyse the formation of all-important ‘big-picture’ theories of brain function and dysfunction, offering novel testable hypotheses which can be examined with the full suite of tools available to modern neuroscience, both within and across micro- and macro-scales. Additionally, the enticing ability to link clinical symptomatology, through characterising trans-nosological molecular dysfunction, to mechanistically informed pharmacotherapy may help finally bring functional imaging closer to clinical reality.

## Conflicts of interest disclosure

All authors have no formal conflicts of interest to declare.
